# Better Together: A Model for Women and LGBTQ Equality in the Workplace

**DOI:** 10.3389/fpsyg.2019.00272

**Published:** 2019-02-20

**Authors:** Carolina Pía García Johnson, Kathleen Otto

**Affiliations:** Faculty of Psychology, Work and Organizational Psychology, Philipps University of Marburg, Marburg, Germany

**Keywords:** diversity, gender equality, gender management, heteronormativity, heterosexism, human resources, intersectionality, LGBTQ

## Abstract

Much has been achieved in terms of human rights for women and people of the lesbian, gay, bisexual, transsexual, and queer (LGBTQ) community. However, human resources management (HRM) initiatives for gender equality in the workplace focus almost exclusively on white, heterosexual, cisgender women, leaving the problems of other gender, and social minorities out of the analysis. This article develops an integrative model of gender equality in the workplace for HRM academics and practitioners. First, it analyzes relevant antecedents and consequences of gender-based discrimination and harassment (GBDH) in the workplace. Second, it incorporates the feminist, queer, and intersectional perspectives in the analysis. Third, it integrates literature findings about women and the LGBTQ at work, making the case for an inclusive HRM. The authors underscore the importance of industry-university collaboration and offer a starters' toolkit that includes suggestions for diagnosis, intervention, and applied research on GBDH. Finally, avenues for future research are identified to explore gendered practices that hinder the career development of women and the LGBTQ in the workplace.

## Introduction

Gender has diversified itself. More than four decades have passed since Bem (1974) published her groundbreaking article on psychological androgyny. With her work, she challenged the binary conception of gender in the western academia, calling for the disposal of gender as a stable trait consistent of discrete categories (Mehta and Keener, 2017). Nowadays, people from the LGBTQ community find safe spaces to express their gender in most developed countries (see ILGA-Europe, 2017). Also, women-rights movements have impulsed changes for the emancipation and integration of women at every social level, enabling them to achieve things barely imaginable before (see Hooks, 2000).

However, there is still a lot to do to improve the situation of women and people from the LGBTQ community (International Labour Office, 2016; ILGA-Europe, 2017). Some actions to increase gender inclusion in organizations actually *conceal inequality* against women, and many problems faced by the LGBTQ originate within frameworks that anti-discrimination policy *reinforce* (see Benschop and Doorewaard, 1998, 2012; Verloo, 2006). For example, the gender equality, gender management, and gender mainstreaming approaches overlook most problems faced by people from the LGBTQ community and from women of color, framing their target stakeholders as white, cisgender, and heterosexual (see Tomic, 2011; Hanappi-Egger, 2013; Klein, 2016). These problems seem to originate in the neoliberalization of former radical movements when adopted by the mainstream (see Cho et al., 2013). This translates into actions addressing sexism and heterosexism that overlook other forms of discrimination (e.g., racism, ableism), resisting an intersectional approach that would question white, able-bodied, and other forms of privilege (see Crenshaw, 1991; Cho et al., 2013; Liasidou, 2013; van Amsterdam, 2013).

The purpose of this paper is to support the claim that gender equality shall be done within a queer, feminist, and intersectional framework. This argument is developed by integrating available evidence on the antecedents and consequences of GBDH against women and people from the LGBTQ community in the workplace. The authors believe that GBDH against these groups has its origin in the different manifestations of sexism in organizations. A model with the antecedents and consequences of GBDH in the workplace is proposed. It considers an inclusive definition of gender and integrates the queer-feminist approach to HRM (Gedro and Mizzi, 2014) with the intersectional perspective (Crenshaw, 1991; McCall, 2005; Verloo, 2006). In this way, it provides a framework for HRM scholars and practitioners working to counteract sexism, heterosexism, and other forms of discrimination in organizations.

## GBDH in the Workplace

GBDH is the umbrella term we propose to refer to the different manifestations of sexism and heterosexism in the workplace. The roots of GBDH are beyond the forms that discriminatory acts and behaviors take, being rather “about the power relations that are brought into play in the act of harassing” (Connell, 2006, p. 838). This requires acknowledging that gender harassment is a technology of sexism, that “perpetuates, enforces, and polices a set of gender roles that seek to feminize women and masculinize men” (Franke, 1997, p. 696). Harassment against the LGBTQ is rooted in a heterosexist ideology that establishes heterosexuality as the superior, valid, and natural form of expressing sexuality (see Wright and Wegner, 2012; Rabelo and Cortina, 2014). Furthermore, women and the LGBTQ are oppressed by the institutionalized sexism that underscores the supremacy of hegemonic masculinity (male, white, heterosexual, strong, objective, rational) over femininity (female, non-white, non-heterosexual, weak, emotional, irrational; Wright, 2013; Denissen and Saguy, 2014; Dougherty and Goldstein Hode, 2016). In addition, GBDH overlaps with other frameworks (e.g., racism, ableism, anti-fat discrimination) that concurrently work to maintain white, able-bodied, and thin privilege, impeding changes in the broader social structure (see Yoder, 1991; Yoder and Aniakudo, 1997; Buchanan and Ormerod, 2002; Acker, 2006; Liasidou, 2013; van Amsterdam, 2013). The next paragraphs offer a definition of some of the most studied forms of GBDH in the workplace.

### Sexual Harassment

Sexual harassment was first defined in its different dimensions as gender harassment, unwanted sexual attention, and sexual coercion (Gelfand et al., 1995). Later, Leskinen and Cortina (2013) focused on the gender-harassment subcomponent of sexual harassment and developed a broadened taxonomy of the term. This was motivated by the fact that legal practices gave little importance to gender-harassment forms of sexual harassment, despite of the negative impact they have on the targets' well-being (Leskinen et al., 2011). Gender harassment consists of rejection or “put down” forms of sexual harassment such as sexist remarks, sexually crude/offensive behavior, infantilization, work/family policing, and gender policing (Leskinen and Cortina, 2013). The concepts of sexual harassment and gender harassment were initially developed to refer to the experiences of women in the workplace, but there is also evidence of sexual and gender harassment against LGBTQ individuals (Lombardi et al., 2002; Silverschanz et al., 2008; Denissen and Saguy, 2014). In addition, studies have shown how gender harassment and heterosexist harassment are complementary and frequently simultaneous phenomena accounting for mistreatment against members of the LGBTQ community (Rabelo and Cortina, 2014).

### Gender Microaggressions

Gender microaggressions account for GBDH against women and people from the LGBTQ community that presents itself in ways that are subtle and troublesome to notice (Basford et al., 2014; Galupo and Resnick, 2016). Following the taxonomy on racial microaggressions developed by Sue et al. (2007), the construct was adapted to account for gender-based forms of discrimination (Basford et al., 2014). Gender microaggressions consist of microassaults, microinsults, and microinvalidations, and although they may appear to be innocent, they exert considerably negative effects in the targets' well-being (Sue et al., 2007; Basford et al., 2014; Galupo and Resnick, 2016). As an example of microassault imagine an individual commenting their colleague that their way of dressing looks unprofessional (because it is not “masculine enough,” “too” feminine, or not according to traditional gender-binary standards). A microinsult is for example when the supervisor asks the subordinate about *who* helped them with their work (which was “too good” to be developed by the subordinate alone). An example of microinvalidation would be if in a corporate meeting the CEO dismisses information related to women or the LGBTQ in the company regarding it as unimportant, reinforcing the message that women and LGBTQ issues are inexistent or irrelevant (for more examples see Basford et al., 2014; Galupo and Resnick, 2016). Because gender is not explicitly addressed in microaggressions, it can be especially difficult for the victims to address the offense as such and act upon them (see Galupo and Resnick, 2016). Hence, they are not only emotionally distressing, but also tend to be highly ubiquitous, belonging to the daily expressions of a determined context (Nadal et al., 2011, 2014; Gartner and Sterzing, 2016).

### Disguised Forms of GBDH

It is also the case that some forms of workplace mistreatment constitute disguised forms of GBDH. Rospenda et al. (2008) found in their US study that women presented higher rates of generalized workplace abuse (i.e., workplace bullying or mobbing). In the UK, a representative study detected that a high proportion of lesbian, gay, and bisexual respondents have faced workplace bullying (Hoel et al., 2017). Specifically, the results indicated that while the bullying rate for heterosexuals over a six-months period was of 6.4%, this number was tripled for bisexuals (19.2%), and more than doubled for lesbians (16.9%) and gay (13.7%) individuals (Hoel et al., 2017). Moreover, 90% of the transgender sample in a US study reported experiencing “harassment, mistreatment or discrimination on the job” (Grant et al., 2011, p. 3). These findings suggest that many of the individuals facing workplace harassment that appears to be gender neutral are actually targets of GBDH. Hence, they experience “*disguised* gender-based harassment and discrimination” (Rospenda et al., 2009, p. 837) that should not be addressed as a gender-neutral issue.

### Intersectional, Queer, and Feminist Approaches in Organizations

In this section, a short introduction to the feminist, queer, and intersectional approaches is given, as they are applied to the analyses throughout this article.

## Feminist Approaches

### In the Beginning There Was Feminism

In the words of bell hooks, “[f]eminism is a movement to end sexism, sexist exploitation, and oppression” (Hooks, 2000, viii). However, feminism can be a movement, a methodology, or a theoretical approach, and it is probably better to talk about *feminisms* than considering it a unitary concept. In this paper, different feminist approaches (see Bendl, 2000) are applied to the analysis. *Gender as a variable* takes gender as a politically neutral, uncontested variable; the *feminist standpoint* focuses on women as a group; and the *feminist poststructuralist* approach searches to deconstruct hegemonic discourses that perpetuate inequality (for the complete definitions see Bendl, 2000).

### Gender Subtext

The gender subtext refers to an approach to the managerial discourse that brings attention to how official speeches of inclusion work to conceal inequalities (Benschop and Doorewaard, 1998). Its methodology -subtext analysis- brings discourse analysis and feminist deconstruction together to scrutiny the managerial discourse and practices in organizations (Benschop and Doorewaard, 1998; Bendl, 2000; Bendl, 2008; Benschop and Doorewaard, 2012).

### Integration and Applications of Feminist Approaches and the Gender Subtext

The gender subtext serves to understand the role that organizational factors play in the occurrence of GBDH. Gender as a variable serves to underscore how the hegemonic definition of gender excludes and otherizes the LGBTQ from HRM approaches to gender equality. The feminist standpoint is applied in this paper as a framework in which two groups—women and the LGBTQ—are recognized in their heterogeneity, and still brought together to search for synergies to counteract sexism as a common source of institutionalized oppression (see Oliver, 1992; Franke, 1997). Finally, the feminist-poststructuralist approach enables conceiving gender as deconstructed and reconstructed, and to apply the subtext analysis to the organizational discourse (see Benschop and Doorewaard, 1998; Monro, 2005).

## Queer Approach

### Queer Theory and Politics

The origins of the queer movement can be traced to the late eighties, when lesbians, gays, bisexuals, and the transgender took distance from the LGBT community as a sign of disconformity with the depoliticization of its agenda (Woltersdorff, 2003). However, the “Queer” label was later incorporated in the broader movement (Woltersdorff, 2003). In terms of queer theory, the most recognized scholar is Judith Butler, whose work *Gender Trouble* (1990) was revolutionary because it made visible the oppressive character of the categories used to signify gender, and insisted in its performative nature (see Butler, 1990; Woltersdorff, 2003).

### Queer Standpoint, the LGBTQ, and HRM

In the presented model, queer theory brings attention to the exclusion of the LGBTQ community from the organizational and HRM speech. This exclusion is observed in the policies and politics supported by the HRM literature and practitioners, as well as in the way the LGBTQ are otherized by their discursive practices (e.g., validating only a binary vision of gender, Carrotte et al., 2016). Although the categories that the queer theory criticizes are applied in this model, its constructed nature is acknowledged (see Monro, 2005). In this way, McCall's (2005) argument in favor of the strategic use of categories for the intersectional analysis of oppression is supported. This analysis is conducted adopting a queer-feminist perspective (Marinucci, 2016) and the intersectional approach.

## Integration of Intersectionality With the Queer and Feminist Approaches

### Origin and Approaches

The concept of intersectionality was initially introduced to frame the problem of double exclusion and discrimination that black women face in the United States (Crenshaw, 1989, 1991). Crenshaw (1991) analyzed how making visible the specific violence faced by black women conflicted with the political agendas of the feminist and anti-racist movements. This situation left those women devoid of a framework to direct political attention and resources toward ending with the violence they were (and still are) subjected to (Crenshaw, 1991). Intersectionality theory has evolved since then, and different approaches exist within it (McCall, 2005). These approaches range from fully *deconstructivist* (total rejection of categories), to *intracategorical* (focused on the differences within groups), to *intercategorical* (exploring the experiences of groups in the intersections), and are compatible with queer-feminist approaches (see Parker, 2002; McCall, 2005; Chapman and Gedro, 2009; Hill, 2009).

The intracategorical approach acknowledges the heterogeneity that exist *within* repressed groups (see Bendl, 2000; McCall, 2005). Within this framework (also called intracategorical complexity, see McCall, 2005), the intersectional analysis emerges, calling for attention to historically marginalized groups, [as in Crenshaw (1989, 1991)]. The deconstructivist view helps to de-essentialize categories as gender, race, and ableness, making visible the power dynamics they contribute to maintain (see Acker, 2006). The intercategorical approach takes constructed social categories and analyzes the power dynamics occurring between groups (McCall, 2005).

### Integration: Queer-Feminist Intersectional Synergy

Applying these complementary approaches helps to analyze how women and people from the LGBTQ community are defined (e.g., deconstructivist approach), essentialized (e.g., deconstructivist and intracategorical approaches), and oppressed by social actors (e.g., intercategorical approach) and institutionalized sexism (e.g., Oliver, 1992; Franke, 1997). It also allows the analysis of the oppression reinforced by members of the dominant group (intercategorical approach), as well as by minority members that enjoy other forms of privilege (e.g., white privilege), and endorse hegemonic values (deconstructivist and intracategorical approaches). In addition, the analyses within the inter- and intra-categorical framework allow approaching the problems faced by individuals in the intersections between sexism, heterosexism, cissexism, and monosexism (e.g., transgender women, lesbians, bisexuals), as well as considering the way classism, racism, ableism, and ethnocentrism shape their experiences (e.g., disabled women, transgender men of color).

### Support for an Integrative HRM Model of GBDH in the Workplace

**Figure 1:**
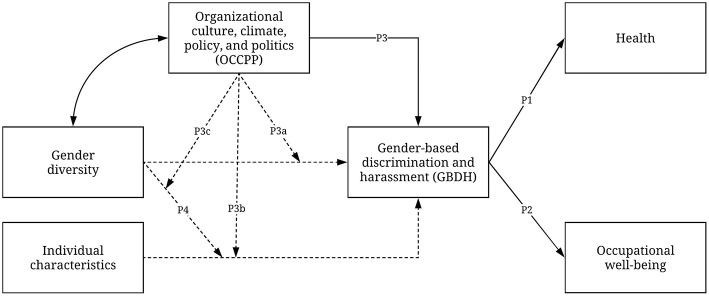
Integrative model of GBDH in the workplace. Continuous paths represent direct relationships. Dashed paths represent fully moderated relationships. The double-ended arrow signals the relationship between gender diversity and OCCPP, which follows a circular causation logic.

This section describes an integrative model of GBDH in the workplace (Figure 1). First, the effects of GBDH on the health and occupational well-being of targeted individuals are illustrated (P1 and P2). Afterwards, the model deals with the direct and moderation effects of organizational climate, culture, policy, and politics (OCCPP) on GBDH in the workplace. OCCPP acts as a “switch” that enables or disables the other paths to GBDH. OCCPP's effects on GBDH are described as: a direct effect on GBDH (P3), the moderation of the relationship between gender diversity and GBDH (P3a), the moderation of the relationship between individual characteristics and GBDH (P3b), and the moderation (P3c) of the moderation effect of gender diversity on the relationship between individual's characteristics and GBDH (P4). In other words, when OCCPP produce environments that are adverse for gender minorities, gender diversity and gender characteristics become relevant to explain GBDH. When OCCPP generate respectful and integrative environments, gender diversity, and gender characteristics are no longer relevant predictors of harassment.

## Consequences of GBDH in the Workplace

### GBDH and Individuals' Health

Evidence suggests that exposure to sexist discrimination and harassment in the workplace negatively affects women's well-being (Yoder and McDonald, 2016; Manuel et al., 2017), and that different forms of sexual harassment can constitute trauma and lead to posttraumatic stress disorder (Avina and O'Donohue, 2002). In their meta-analysis (*N* = 89.382), Chan et al. (2008) found a negative relationship between workplace sexual harassment, psychological health, and physical health conditions. Regarding the LGBTQ at work, Flanders (2015) found a positive relationship between negative identity events, microaggressions, and feelings of stress and anxiety among a sample of bisexual individuals in the US. This is consistent with Galupo and Resnick's (2016) results about the negative effects of microaggressions for the well-being of lesbian, bisexual, and gay workers. In another study, Seelman et al. (2017) found that microaggressions and other forms of gender discrimination relate to lowered self-esteem and increased stress and anxiety in LGBTQ individuals, with the most negative effects reported by the transgender. In a study among gay, lesbian, and bisexual emerging adults in the US, exposure to the phrase “that's so gay” related to feelings of isolation and physical health symptoms as headaches, poor appetite, and eating problems (Woodford et al., 2012). In the literature on gender discrimination, Khan et al. (2017) found that harassment relates to depression risk factors among the LGBTQ. Finally, according to Chan et al. (2008) meta-analysis, targets of workplace sexual harassment suffer its detrimental job-related, psychological, and physical consequences regardless of their gender.

*Proposition P1: GBDH negatively affects women and LGBTQ individuals' health in the workplace*.

### GBDH and Occupational Well-Being

Occupational well-being refers to the relationship between job characteristics and individuals' well-being (Warr, 1990). It is defined “as a positive evaluation of various aspects of one's job, including affective, motivational, behavioral, cognitive, and psychosomatic dimensions” (Horn et al., 2004, p. 366). It has a positive relationship with general well-being (Warr, 1990) and work-related outcomes like task performance (Devonish, 2013; Taris and Schaufeli, 2015).

There is robust evidence on the negative effects of GBDH on indicators of occupational well-being, such as overall job satisfaction, engagement, commitment, performance, job withdrawal, and job-related stress (Stedham and Mitchell, 1998; Lapierre et al., 2005; Chan et al., 2008; Cogin and Fish, 2009; Sojo et al., 2016). Its negative effects have been reported among women (Fitzgerald et al., 1997), gay and heterosexual men (Stockdale et al., 1999), lesbians (Denissen and Saguy, 2014), and transgender individuals (Lombardi et al., 2002), to name some.

*Proposition P2: GBDH negatively affects the occupational well-being of women and people from the LGBTQ community in the workplace*.

## Antecedents of GBDH in the Workplace

### Direct Effect of OCCPP on GBDH

In the next lines, the direct effects of OCCPP on GBDH against women and people from the LGBTQ community are explored, supporting the next proposition of this model.

*Proposition P3: OCCPP affect the incidence of GBDH against women and the LGBTQ*.

#### Organizational Culture and GBDH

Organizational culture refers to the shared norms, values, and assumptions that are relatively stable and greatly affect the functioning of organizations (Schein, 1996). The most plausible link between organizational culture and GBDH seems to be the endorsement of sexist beliefs and attitudes. This is supported by evidence that sexism endorsement encourages GBDH attitudes and behavior (see Pryor et al., 1993; Fitzgerald et al., 1997; Stockdale et al., 1999; Stoll et al., 2016). The literature on sexism has mainly adopted a binary conception of gender (see Carrotte et al., 2016). However, the last decade more research has focused on heterosexism and anti-LGBTQ attitudes, uncovering their negative effects in the lives of LGBTQ individuals.

#### Sexism Against Women

Scholars focusing on sexism against women have categorized it in different ways. *Old-fashioned sexism* refers to the explicit endorsement of traditional beliefs about women's inferiority (Morrison et al., 1999). *Modern* and *neo sexism* define the denial of gender inequality in society and resentment against measures that support women as a group (Campbell et al., 1997; Morrison et al., 1999). *Gender-blind sexism* refers to the denial of the existence of sexism against women (Stoll et al., 2016). *Benevolent sexism* defines the endorsement of an idealized vision of women that is used to reinforce their submission (Glick et al., 2000). Finally, *ambivalent sexism* is the term for the endorsement of both hostile and “benevolent” sexist attitudes (Glick and Fiske, 1997, 2001, 2011).

#### Sexism Against the LGBTQ

Sexism directed against the LGBTQ takes different forms, that can be also held by members of the LGBTQ community, as the evidence about biphobia and transphobia points out (see Vernallis, 1999; Weiss, 2011). *Heterosexism* is the endorsement of beliefs stating that heterosexuality is the normal and desirable manifestation of sexuality, while framing other sexual orientations as deviant, inferior, or flawed (see Habarth, 2013; Rabelo and Cortina, 2014). *Monosexism* and *biphobia* refer to negative beliefs toward people that are not *monosexual*, namely, whose sexual orientation is not defined by the attraction to people from only one gender (see Vernallis, 1999). *Cissexism* (also *transphobia*) refers to “an ideology that denigrates and subordinates trans^*^ people because their sex and gender identities exist outside the gender binary. Transgender people are thus positioned as less authentic and inferior to cisgender people” (Yavorsky, 2016, p. 950). Hence, transgender individuals experience concurrently sexism, heterosexism, and cissexism/transphobia in their workplaces (see Yavorsky, 2016).

#### Organizational Climate and GBDH

Organizational climate reflects the “social perceptions of the appropriateness of particular behaviors and attitudes [in an organization]” (Sliter et al., 2014). There is evidence linking organizational climate with workplace harassment (Bowling and Beehr, 2006), sexual harassment (Fitzgerald et al., 1997, p. 578), and gender microaggressions (Galupo and Resnick, 2016).

*Diversity climate* is “the extent to which employees perceive their organization to be supportive of underrepresented groups, both in terms of policy implementation and social integration” (Sliter et al., 2014). Hence, a gender-diversity climate reflects the employees' perceptions of their workplace as welcoming and positively appreciating gender differences (Jansen et al., 2015). It has been associated with an increased perception of inclusion by members of an organization, buffering the negative effects of gender dissimilarity (i.e., gender diversity) between individuals in a group (Jansen et al., 2015). Sliter et al. (2014) found a negative relationship between diversity climate perceptions and conflict at work. Also, it has been suggested that it plays a crucial role for workers' active support of diversity initiatives, which is determinant for their successful implementation (Avery, 2011). A similar construct, *climate for inclusion* has also shown to be a positive factor in gender-diverse groups, protecting against the negative effects of group conflict over unit-level satisfaction (Nishii, 2013).

*Heterosexist climate* refers to an organizational climate in which heterosexist attitudes and behaviors are accepted and reinforced, propitiating GBDH against the LGBTQ (see Rabelo and Cortina, 2014; Galupo and Resnick, 2016). For example, Burn et al. (2005) conducted a study using hypothethical scenarios to test the effects of indirect heterosexism on lesbians, gays, and bisexuals. The participants of their study reported that hearing heterosexist comments would be experienced as an offense, affecting their decision to share information about their sexual orientation (Burn et al., 2005). In addition, it has been found that LGBTQ-friendly climates (hence, low in heterosexism), can have a positive impact on the individual and organizational level (Eliason et al., 2011). Examples of positive outcomes are reduced discrimination, better health, increased job satisfaction, job commitment (Badgett et al., 2013), perceived organizational support (Pichler et al., 2017), and feelings of validation for lesbians that become mothers (Hennekam and Ladge, 2017).

#### Workplace Policy and GBDH

Workplace policy plays an important role in the incidence of GBDH. Finally, evidence shows that policy affects the extent to which the work environment presents itself as LGBTQ-friendly, influencing the experience of LGBTQ individuals at work (Riger, 1991; Eliason et al., 2011; Döring, 2013; Dougherty and Goldstein Hode, 2016; Galupo and Resnick, 2016; Gruber, 2016). Eliason et al. (2011) found that inclusive language, domestic partner benefits, child-care solutions, and hiring policies are relevant for the constitution of a gender-inclusive work environment for the LGBTQ. Calafell (2014) wrote about how the absence of policy addressing discrimination against people with simultaneous minority identities (e.g., queer Latina) contributes to cover harassment against them. Galupo and Resnick (2016) found that weak policy contributes to the incidence of microaggressions against people from the LGBTQ community. Some of the situations they found include refusal of policy reinforcement, leak of confidential information, and refusal to acknowledge the gender identity of a worker (Galupo and Resnick, 2016). Moreover, existent policy may serve to reinforce inequalities if its discourse is based on power binaries (e.g., rational/masculine vs. emotional/feminine) that discredit, oppress, and marginalize minority groups (Riger, 1991; Dougherty and Goldstein Hode, 2016). For example, Peterson and Albrecht (1999) analyzed maternity-policy and found how discourse is shaped to protect organizational interest at the cost of the precarization of women's conditions in organizations. Finally, it is very important to address the mishandling of processes and backlash after GBDH complaints are filed, since they keep targets of harassment from seeking help within their organizations (see Vijayasiri, 2008).

#### Organizational Politics and GBDH

Organizations are political entities (Mayes and Allen, 1977). In the workplace, power, conceived as access to information and resources, is negotiated through political networks embedded in communication practices (Mayes and Allen, 1977; Mumby, 2001; Dougherty and Goldstein Hode, 2016). These communication practices operate within power dynamics in which the majority group sets the terms of the discussion and frames what is thematized (Mumby, 1987, 2001). Since gender affects the nature of these power relations, the effects of politics in gender issues and of gender issues in politics must be considered.

### Full Moderation of OCCPP of the Relationship Between Gender Diversity and GBDH

Gender diversity refers to heterogeneity regarding gender characteristics of individuals in an organization. Broadly, an organization in which most workers are cisgender, male, and heterosexual would be low in gender diversity, and one in which individuals are evenly distributed in terms of their gender identity, sexual orientation, and gender expression, would be high on gender diversity. In this section, the moderation effect of OCCPP on the relationship between gender diversity and GBDH is discussed to support the next proposition of the model.

*Proposition P3a: The relationship between gender diversity and GBDH is fully moderated by OCCPP. When OCCPP propitiate a hostile environment for gender minorities, low gender diversity will lead to high GBDH. When OCCPP propitiate a context of respect and integration of gender minorities, low gender diversity will not lead to higher GBDH*.

#### Male-Dominated Workplace

In male-dominated organizations, a hypermasculine culture is predominant, male workers represent a numerical majority, and most positions of power are occupied by men (e.g., Carrington et al., 2010). These organizations present an increased frequency and intensity of GBDH against women, men who do not do gender in a hypermasculine form, and individuals from the LGBTQ community (Stockdale et al., 1999; Street et al., 2007; Chan, 2013; Wright, 2013). Women in a male-dominated workplace may be confronted with misogyny at work (Denissen and Saguy, 2014), becoming targets of more intense and frequent GBDH as they depart from the policed gender-rule that demands them to behave feminine, submissive, and heterosexual (Berdahl, 2007). Women refusing sexual objectification in these contexts may become targets of serious forms of mistreatment, with the case that certain women “—including lesbians and those who present as butch, large, or black—may be less able to access emphasized femininity as a resource and thus [become] more subject to open hostility” (Denissen and Saguy, 2014, p. 383). In other words, the more they depart from the sexist and heteronormative standard, the worse is the mistreatment they will face. At the same time, the strategies some women apply to avoid hostility have a high cost for their identity and validation at work, as pointed by Denissen and Saguy (2014, p. 383),

the presence of lesbians threatens heteronormativity and men's sexual subordination of women […] [b]y sexually objectifying tradeswomen, tradesmen, in effect, attempt to neutralize this threat. While tradeswomen, in turn, are sometimes able to deploy femininity to manage men's conduct and gain some measure of acceptance as women, it often comes at the cost of their perceived professional competence and sexual autonomy and—in the case of lesbians—sexual identity.

However, GBDH is not only directed to women in hypermasculine contexts, as suggested by Denissen and Saguy (2014), who observed that “tradesmen unapologetically use homophobic slurs to repudiate both homosexuality and femininity (in men)” (Denissen and Saguy, 2014, p. 388). Hence, men working in a male-dominated context are also expected to perform hegemonic masculinity, being punished when they do not comply. This leaves men who do not present dominant traits, that are feminine, or that are not heterosexual, at risk of becoming targets of GBDH (Franke, 1997; Stockdale et al., 1999; Carrington et al., 2010).

#### Female-Dominated Workplace

Female-dominated workplaces are those where women represent a numeric majority. It has been suggested that in these contexts (e.g., nursing) women with care responsibilities can find more tools to balance work-family schedules (Caroly, 2011), and face less harassment (Konrad et al., 2010). However, evidence about heterosexism and harassment against people from the LGBTQ community uncovers heteronormativity in female-dominated workplaces (e.g., among nurses, see Eliason et al., 2011). For example, an experiment about discrimination of gays and lesbians in recruitment processes showed that while gay males were discriminated in male-dominated occupations, lesbians were discriminated in female-dominated ones (Ahmed et al., 2013).

#### Representation of the LGBTQ in the Workplace

At the moment this paper is being written, the authors have not found research that specifically targets LGBTQ-dominated organizations. There is evidence suggesting that having more lesbian, gay, and non-binary coworkers contributes to the development of LGBTQ-friendly workplaces (Eliason et al., 2011). In addition, evidence supports the positive effects of having LGBTQ leaders that advocate for the respect and integration of LGBTQ individuals in organizations (Moore, 2017).

#### Gender Diversity, Tokenism, Glass Escalator, and GBDH

When gender-minority individuals are pioneers entering a gender-homogeneous workplace, they face a heightened probability of experiencing tokenism (Maranto and Griffin, 2011). Tokenism refers to the performance pressures, social isolation, and role encapsulation that individuals from social minorities face in organizations in which they are underrepresented numerically (Yoder, 1991). Gardiner and Tiggemann (1999) conducted a study comparing the effects of male- and female-dominated work environments on individuals' well-being and tokenism experiences. They found that women, in comparison to men, experience the highest levels of tokenism and discrimination in male-dominated sectors, and that they endure more pressure than men, even in *female*-dominated contexts (Gardiner and Tiggemann, 1999). There is also an increasing number of reports on the experiences of tokenism by the LGBTQ (LaSala et al., 2008; Colvin, 2015) and research on how to hinder the negative consequences of tokenism against them in organizations (Davis, 2017; Nourafshan, 2018). The fact that men in female- dominated work settings report less levels of pressure than women in male dominated workplaces is compatible with Yoder's (1991) conception of tokenism as the oppression of social-minority members who are simultaneously a numerical minority. Because white men are a social majority, they do not experience the negative effects of tokenism when they are underrepresented numerically. Actually, evidence on the *glass escalator* effect shows that white men experience advantages when they enter female-dominated fields (Williams, 1992, 2013, 2015; Woodhams et al., 2015). However, tokenism might be also present in female-dominated settings, as can be inferred from studies on LGBTQ experiences in women-dominated professions (Eliason et al., 2011; Ahmed et al., 2013). Moreover, research in the US suggests that female CEOs tend to advance policies related to domestic-partner benefits and discrimination against women, but not necessarily advocate for a wider range of LGBTQ-inclusion policies (Cook and Glass, 2016).

#### Gender Diversity, Contradictions, and the Role of OCCPP

The evidence on the effects of gender diversity in organizations is not free of contradictions. It has been found that the integration of male coworkers in female-dominated workplaces increases conflict between women (Haile, 2012), and that as the proportion of male doctors in workgroups increases, the same happens with sexual harassment against female doctors (Konrad et al., 2010). If taken together, it makes sense to consider an interaction of OCCPP and gender diversity to explain GBDH. In other words, it seems that gender diversity alone is not enough to end GBDH in the workplace, but can interact in a positive way with organizational factors to diminish conflict and GBDH (see Nishii, 2013). White, middle class, cisgender, heterosexual men would most likely not be targeted for GBDH in female-dominated contexts, since they are not a social minority, rather benefiting from their underrepresentation (see Williams, 1992). Finally, it is expected that gender diversity and OCCPP present a circular causation (see double-ended arrow in Figure 1), so that a higher representation of a particular minority group will traduce into OCCPP that promote inclusion for that group. At the same time, an organization whose OCCPP invites to respect and integrate gender minorities will attract more women and LGBTQ individuals (see Bajdo and Dickson, 2001; Moore, 2017).

### OCCPP Full Moderation of the Relationship Between Individuals' Characteristics and GBDH

Individuals' gender characteristics intersect with race, class, ethnicity, and disability configuring complex identities and dynamics that affect individuals' experience of inequality in organizations (see Oliver, 1992; Acker, 2006; Verloo, 2006; Cunningham, 2008; Ericksen and Schultheiss, 2009; Cho et al., 2013; Donovan et al., 2013; Liasidou, 2013; Wright, 2013; Calafell, 2014; Moodley and Graham, 2015; Senyonga, 2017). In other words, it is difficult to isolate causes for exclusion, since they derive from complex power dynamics that shape individuals' experience. It was mentioned above that women and the LGBTQ tend to be more targeted for GBDH than white heterosexual men. However, it is in sexist organizational contexts that gender characteristics are made salient to propitiate GBDH.

*Proposition P3b: The link between individuals' gender characteristics and GBDH in the workplace is fully moderated by OCCPP. This means that in a context of sexist OCCPP, individuals with gender-minority status will experience more GBDH. In contexts in which OCCPP propitiate respect and integration of gender minorities, GBDH will be low*.

In other words, if the organizational context is tolerant of GBDH, harassment will occur based on individuals' sex, gender identity, sexual orientation, gender expression, or an intersection of those (Crenshaw, 1991; Pryor et al., 1993; Franke, 1997; Stockdale et al., 1999; Galupo and Resnick, 2016). Some examples of how gender characteristics are used as grounds for GBDH are described in the following lines.

*Sex assigned at birth* refers to the gender category assigned to individuals according to their physical characteristics at birth (ILGA-Europe, 2016). At the moment, the intersex category for those whose physical characteristics do not match the binary conception of gender at birth is not officially recognized in many countries (ILGA-Europe, 2016).

*Gender identity* is the “deeply felt internal and individual experience of gender, which may or may not correspond with the sex assigned at birth” (International Commission of Jurists, 2009, p. 6). Despite the claims to adopt inclusive conceptions of gender, organizations continue to direct their gender-equality programs to white cisgender women, excluding the transgender and genderqueer (see Carrotte et al., 2016; Galupo and Resnick, 2016).

*Gender expression* is the way people handle their physical or external appearance so that it reflects their gender identity (European Union Agency for Fundamental Rights, 2014). In highly sexist organizations, gender policing and harassment is directed against less gender-conforming individuals (e.g., Stockdale et al., 1999; Wright, 2013).

*Sexual orientation* refers to the “person's capacity for profound affection, emotional and sexual attraction to, and intimate and sexual relations with, individuals of a different gender or the same gender or more than one gender” (ILGA-Europe, 2016, p. 180). It is often the case that family policy in organizations consider only workers whose families are conformed by heterosexual couples and their children (e.g., Galupo and Resnick, 2016). This excludes those who are in same-sex or non-monosexual partnerships and families, sending the message that they are “different,” abnormal, or unnatural (see Galupo and Resnick, 2016). There is evidence that gender-exclusive language (using *he* and *his* instead of gender-inclusive forms) negatively affects the sense of belongingness, identification, and motivation of women in work settings (Stout and Dasgupta, 2011). In the same way, the exclusion of people with non-binary or non-heterosexual gender characteristics in the organizational discourse makes them experience feelings of exclusion and otherization (Carrotte et al., 2016).

### Double Moderation of OCCPP: Its Effects on the Moderation of Gender Diversity of the Relationship Between Individuals' Characteristics and GBDH

Considering the literature on tokenism, gender characteristics (e.g., transgender) are expected to be a relevant predictor of GBDH if there is a reduced number of people with *those* characteristics in the organization (i.e., low gender diversity). Also, it is expected that this relationship will only take place in those situations in which the OCCPP propitiate a discriminatory and harassing environment for gender minorities.

*Proposition P3c and P4: When OCCPP propitiate a discriminatory and harassing environment for gender minorities, women and the LGBTQ will experience more GBDH in a context low in gender diversity. If the OCCPP configure an environment that is inclusive and respectful of gender minorities, a low gender diversity will not lead to GBDH against women and the LGBTQ in that organization*.

## Recommendations for Academics and Practitioners

### Need for Industry-University Collaborations: From the Lab to the Field

Research that emerges from industry-university collaboration (IUC) is needed to better understand and counteract GBDH. Porter and Birdi (2018) identified twenty-two factors for a successful IUC. Some of these factors are: capacity of the stakeholders to enact change, a clear and shared vision, trust between the actors, and effective communication (Porter and Birdi, 2018). Rajalo and Vadi (2017) developed a model of IUC, according to which success is more likely when preconditions from the involved partners (i.e., academics and practitioners) match. These preconditions are explained in terms of *absorptive capacity* (ability to process and incorporate new information), and *motivation to collaborate* (Rajalo and Vadi, 2017). In other words, those involved in IUC need top management support, economic resources, a shared vision of gender equality, trust in each other, effective communication channels, and high motivation to collaborate. It is not a simple endeavor, but it is a necessary and possible one (see Porter and Birdi, 2018).

In collaborations, scholars and practitioners have the opportunity to work together in the design, development, implementation, and follow-up of HRM strategies. This must be done ensuring that projects are appropriate for each organization, and that the raised information is suitable for research purposes. Evidence on IUC spillover points out that firms and academics benefit from these collaborations (see Jensen et al., 2010). In the case of HRM, scholars can gain access to samples that are difficult to reach and economic resources to finance their research, while practitioners benefit from the academic expertise (see Jensen et al., 2010). In the context of gender equality, this can be useful to develop and implement evidence-based procedures to counteract GBDH (see Briner and Rousseau, 2011). To build the networks necessary for such collaborative alliances, public and private initiative must be taken (see Lee, 2018). Congresses and events that approach gender issues in organizations and aim to build bridges between the industry and the academia can offer opportunities for collaboration to occur. Finally, practitioners must gain awareness of gender issues in the workplace, and organizational-feminist scholars should write and reach for the practitioner audience as well.

### A Small Help to Begin With: The Gender-Equality Starters' Toolkit

We know that for practitioners and researchers that are not familiarized with the poststructuralist, intersectional, queer-feminist theories, our recommendations may sound quite cryptic. For this reason, we developed a very simplified starters' toolkit (Table 1). In its “HRM diagnose” section, we suggest ways to develop a first diagnose of the organization in relation to gender issues. The “HRM interventions” section refers to actions that can be taken in case further intervention is needed. In the “applied-research” section, we provide applied-research ideas to better understand GBDH and develop evidence-based tools for HRM. Finally, in the “references and resources” section we include references that support and complement the suggestions provided. Each row of the toolkit refers to one of the components of our model (health and occupational well-being were grouped together). As mentioned, the aim of this toolkit is to provide material for a first approach to GBDH in organizations, and inspire those interested in conducting applied research on GBDH in the workplace.

**Table 1:** Recommendations for HRM practitioners and applied researchers: a starters' toolkit.

	**HRM diagnose**	**HRM interventions**	**Applied-research: development of resources for practitioners and academics**	**References and resources**
GBDH	1. Measure the incidence of GBDH considering its different forms 2. Conduct interviews and/or focus groups to better interpret the obtained results when in doubt 3. If available, check the record from the sexual harassment helpline from the organization (or equivalent)	1. Train the whole organization on GBDH, focusing on the forms of GBDH that are most prevalent (e.g., microaggressions) 2. Approach those areas where the incidence of GBDH was reported 3. Make sure that GBDH complaints have been addressed and that they are being handled fairly 4. Pay special attention to “stuck” processes and follow-up for adequate closure 5. Address disguised forms of GBDH according to the OCCPP they relate to. For example, develop a plan for cultural change to counteract sexism (culture), implement training and coaching programs for leaders (climate), analyze policy for career development (policy), generate incentives for leaders to mentor and impulse the development of women and the LGBTQ in the workplace (politics)	1. Psychometric validation of scales to measure GBDH 2. Criterion validation of diagnose measures of GBDH 3. Development and validation of measures that address different forms of GBDH 4. Development and validation of standardized training techniques to address GBDH in the workplace 5. Pre-post evaluation of the effects of training interventions to reduce GBDH (e.g., applying the ADDIE model, see Molenda, 2003)	1. Sexual harassment in the workplace (Fitzgerald et al., 1997, 1999) 2. Gender harassment (Leskinen and Cortina, 2013) 3. Gender heterosexist harassment (Rabelo and Cortina, 2014) 4. Homonegative microaggressions (Wegner and Wright, 2016) 5. Microaggressions against women (Owen et al., 2010; Basford et al., 2014) 6. Bullying at work/mobbing (Einarsen et al., 2009) 7. Handling and preventing sexual harassment in the workplace (Kleiner and Takeyama, 1998; McDonald et al., 2015; Newman, 2018) 8. Task allocation processes (de Pater et al., 2009) 9. Illegitimate tasks' effect on ERI, moderated by gender (Omansky et al., 2016)
Organizational climate	1. Apply GBDH-relevant measures of organizational climate 2. Evaluate if leaders promote a climate of respect, diversity, and inclusion 3. Conduct qualitative techniques (e.g., interviews, focus groups) to clarify the meaning of the obtained data whenever necessary	1. Develop action plans for those areas with higher levels of organizational heterosexist climate to promote a climate of respect and inclusion 2. Implement coaching and learning programs for leaders to promote a climate of respect and inclusion 3. Follow-up on the implementation and success of these actions and repeat climate measurements periodically (e.g., once a year)	1. Psychometric and criterion validation of GBDH-relevant measures of organizational climate 2. Longitudinal analysis of the effects of leadership and organizational-climate interventions to test their effectiveness	1. Psychological climate for sexual harassment (Estrada et al., 2011) 2. Diversity climate (Mor Barak et al., 1998) 3. Climate for inclusion (Nishii, 2013) 4. LGBT organizational-climate inventory (Liddle et al., 2004)
Organizational culture	1. Apply qualitative and quantitative techniques to measure the endorsement of hetero-sexist values in the organization 2. Determine cultural elements (Schein, 1990, 1996) that contribute to reinforce a hetero-sexist culture 3. Engage key members in the organization (visible and/or powerful) to serve as champions to impulse cultural change 4. Identify excluding and otherizing communication practices	1. Develop an action plan for cultural change 2. Start changing the HRM elements that contribute to reinforce heterosexism (e.g., heterosexist communication material, events, official communicates, exclusive language etc.), then reach for the rest of the organization. Cunningham (2008) underscores the importance of political, social, and functional pressures that can be utilized to question the legitimacy of sexism in organizations to impulse cultural change 3. Implement a “Champions” program for gender diversity and inclusion, making visible top-leaders' advocacy for these issues 4. Implement gender-validating communication practices in all organizational-communication instances	1. Validate methods for cultural change to apply in the organizational setting 2. Determine what forms of heterosexism are most prevalent in determined organizational settings 3. Develop studies to uncover forms in which organizational culture manifests heterosexism 4. Develop and share experiences on implementing inclusive and gender-validating communication practices	1. Heterosexism (Rabelo and Cortina, 2014) 2. Modern sexism and old-fashioned sexism (Morrison et al., 1999) 3. Hostile and benevolent sexism (Glick and Fiske, 2001) 4. Neosexism (Campbell et al., 1997) 5. Antecedents of deinstitutionalization that cause cultural change in organizations (Oliver, 1992) 6. Application of the antecedents of deinstitutionalization (Cunningham, 2008) 7. Organizational advocacy and championship for cultural change (Cameron and Green, 2009) 8. Gendered communication practices (Stout and Dasgupta, 2011; Carrotte et al., 2016)
Organizational policy	1. Check for availability and applicability of policy addressing GBDH, partner benefits, and parenthood, among others 2. Analyze available procedures in case GBDH occurs 3. Analyze the organizational history regarding policy compliance, policy reinforcement, recurrence of harassment, etc.	1. Develop policy whenever it is missing 2. Revise the way policy has been interpreted to solve cases of GBDH 3. Deconstruct policy subtext to understand the operating gender-power dynamics 4. Develop communication campaigns to inform all organization members about the available policy and procedures	1. Document and share experiences on policy deconstruction and policy development 2. Make visible the ways by which gender-subtext operates within organizational policy	1. On the effects of policy and considerations for its development (Hirsh and Cha, 2016) 2. On analyzing and interpreting gender subtext and applying a feminist-queer-intersectional approach (Bendl, 2000, 2008) 3. On applying queer theory to unveil heteronormative essentialist definitions of gender in organizations (Bendl et al., 2008) 4. Deconstruction of sexual harassment policy (Dougherty and Goldstein Hode, 2016) 5. Deconstruction of maternity-leave policy (Peterson and Albrecht, 1999; Buzzanell and Liu, 2005) 6. Deconstruction of the predominant discourse in the Swedish nursing profession (Dahlborg-Lyckhage and Pilhammar-Anderson, 2009)
Organizational politics	Answer these questions to analyze how power is distributed in the organization: 1. How many women and LGBTQ hold positions of power? How strong are the glass and lavender ceilings? 2. What career-development opportunities are available for women and the LGBTQ? 3. In case certain minority groups are underrepresented in the organization in general, what is the discourse that accounts for that? 4. What is the discourse that accounts for the underrepresentation of certain minority groups in power positions (if that is the case)? 5. Which groups of people are excluded (or feel excluded) from formal and informal decision-making processes? 6. What are the procedures for recruitment, training, and promotion? To what extent are they designed to ensure fairness and accountability? 7. Which groups of people are excluded (or feel excluded) from formal and informal meetings and events?	1. Develop mentoring programs for women and the LGBTQ 2. Remove barriers for minority-group participation in formal and informal events (e.g., early instead of late meetings, invite partners rather than wifes/husbands, use inclusive language) 3. Revise and develop procedures for recruitment, training, and promotion to ensure fairness and accountability 4. Hold line managers and executives accountable for their recruitment, development, and promotion decisions (see Hirsh and Cha, 2016) 5. Remove or redesign events that paramount heterosexist values (e.g., Christmas party with an heterosexist comedian; women portrayed in sexualized ways [e.g., as models in stretch clothes handling the prizes to the awarded men])	1. Test, document, and share effectiveness of mentoring programs 2. Share case studies on political-change management 3. Develop indicators of distribution of power inside organizations 4. Share HRM best-practices to ensure equal opportunity for people of all gender characteristics and other minorities as well	1. Mentoring and politics for women in the academia (Gibson, 2006; Dashper, 2018) 2. Testimony of the researchers' positive mentorship experience, and their reflections on the importance of mentors' awareness of gender issues when mentoring women (Ali and Coate, 2012) 3. Mentoring for LGBTQ youth (McAllister et al., 2009) 4. On the positive effects of policy designed to increase accountability in recruitment, hiring, and promotion processes for the representation of minorities at the managerial level (Hirsh and Cha, 2016) 5. On power, politics, and gender (Mumby, 1996, 2001; Bendl, 2000; Bendl et al., 2008)
Individuals' health and occupational well-being	1. Raise data on individuals' occupational well-being (e.g., job satisfaction, job commitment, organizational engagement, burnout) 2. Raise data on individuals' health, sick absence, and sick presenteeism 3. Check for intergroup differences (e.g., t-tests/ANOVA) controlling for gender characteristics that may signal the presence of sexist OCCPP and/or GBDH 4. Check for intergroup differences involving people in the intersections of minority identities	1. Identify areas that present significantly higher rates of sick absence, sick presenteeism, health problems, and lower occupational well-being for women and/or the LGBTQ. Implement actions to improve the OCCPP and counteract possible GBDH (see above). Check for additional problems that affect people in the intersections of minority identities and develop actions to address them accordingly 2. Repeat measurements periodically (e.g., once a year) for follow-up and to evaluate the effectiveness of the interventions	1. Test criterion-validity (utilizing health and occupational well-being indicators) of diagnose instruments and methods to reduce GBDH in organizations 2. Raise evidence on women's and LGBTQ's health and occupational well-being in the workplace 3. Raise evidence to make visible the problems faced by individuals in the intersection of multiple minority identities	1. Meta-analysis on the antecedents and consequences of sexual harassment in the workplace (Chan et al., 2008) 2. Workplace harassment and morbidity among US adults (Khubchandani and Price, 2015) 3. Work conditions and mental health of dual-earner gay/lesbian parents (Goldberg and Smith, 2013) 4. On the influence of workplace policy on LGBT well-being in the workplace (Lloren and Parini, 2017) 5. Quantitative methodology in intersectional research (Else-Quest and Hyde, 2016a,b).

## A Change of Perspective: Looking at the Organization with Queer-Feminist Lens

### Change Organizational Politics, Change the Organization

Organizational politics result from the interplay of discursive practices and power negotiations, and refer to who and how is determining the terms of these negotiations (Mumby, 1987, 2001). To understand organizational politics, the hegemonic discourse has to be analyzed utilizing deconstructive lens that uncover the operating power dynamics (e.g., Benschop and Doorewaard, 1998; Dougherty and Goldstein Hode, 2016). In other words, when deconstructing the organizational discourse, the researcher or practitioner analyzes both the content and structural elements of the particular text (see Peterson and Albrecht, 1999; Buzzanell and Liu, 2005). Organizational-text examples are: the sexual harassment policy of the organization, brochures from the last organizational-change campaign, the transcript of interviews on gender issues, the chart of values of the firm. The analysis of this material allows to observe the way gender issues are approached and defined (or not approached nor defined), to develop a first diagnose and lines of action (for an example see Dougherty and Goldstein Hode, 2016). Some questions that may help in the analysis are:

*How is gender defined?* (Whose gender is [not] validated?),
*What actions or behaviors are constitutive of GBDH in this organization?* (What forms of aggression and discrimination are hence allowed?),
*What are the procedures if action is to be taken?* (What is left out of procedure leaving space for leaks or inadequacies?), and
*What is the organizational history in relation to GBDH claims?* (Who has enjoyed impunity? Whose claims are [not] listened to?).

For example, the researcher or practitioner may realize that the sexual-harassment policy of a particular organization refers to cisgender individuals only. Moreover, it may be that this policy defines GBDH as harassment of men against women, excluding same-sex sexual harassment (see Stockdale et al., 1999). Furthermore, it may become evident that this policy is framed in a discourse of binary logics that serve to blame the victims and victimize harassers (see Dougherty and Goldstein Hode, 2016). Finally, after a follow-up of archived organization's processes, it may come out that harassers have historically enjoyed impunity (see Calafell, 2014). This initial analysis might be useful to develop a plan for change. Continuing with the example, this policy may be redefined so that it adopts an integrative conception of gender. In addition, it can be adapted to include cases of same-sex sexual harassment. It can be also reframed using a discourse that allows fairness for all parties involved. Finally, cases from the past may be analyzed to avoid committing old mistakes in the future, and if some of these cases are recent, rectification may be considered.

### Reading Between the Lines: Disguised Forms of GBDH

#### Bullying and Mobbing as Disguised GBDH

We argue that at least some workplace mistreatment that appears as “gender neutral” is actually gendered. Available evidence points to a higher frequency of bullying/mobbing against women and the LGBTQ in the workplace (Rospenda et al., 2008, 2009; Grant et al., 2011; Hoel et al., 2017). Hence, once data on workplace mistreatment is raised, it is advisable to evaluate gender disparities (e.g., statistically comparing means) that may point to cases of disguised GBDH. The importance of addressing disguised GBDH (i.e., “sexist” mobbing and bullying) lies on solving the problem (i.e., mistreatment) at its roots. According to our model, if sexist OCCPP are intervened and changed, their consequences (i.e., overt and disguised forms of GBDH) should disappear.

#### Disguised GBDH at the Task Level

We also believe that disguised GBDH might take place through task allocation processes. In other words, it may be that the processes of task allocation are such that they keep gender minorities away from career-development opportunities. Evidence signaling that women receive less challenging tasks that are relevant for career development suggests that the process of task allocation is not gender neutral (de Pater et al., 2009). There is also research on the effects of illegitimate tasks that suggests that their assignation to individuals in organizations may be gendered (Omansky et al., 2016). Illegitimate tasks are perceived as unreasonable and/or unnecessary by the person that undertakes them, and constitute a task-level stressor (Semmer et al., 2010, 2015). It was found that illegitimate tasks exert a stronger negative effect on perceptions of effort-reward imbalance (ERI) among male than female professionals (Omansky et al., 2016). One explanation is that women are socialized to undertake these tasks, which is why they feel less disrupted by them (Omansky et al., 2016). However, if this causes women to undertake more illegitimate tasks than men, that might bring negative consequences for their occupational development and well-being. Available evidence shows no gender differences in the reports of illegitimate tasks between women and men (see Semmer et al., 2010, 2015; Omansky et al., 2016). However, it is unclear if this is because women do not perceive the tasks they undertake to be illegitimate, or if there is no difference *de facto*. To our knowledge, there is no evidence on illegitimate tasks assigned to LGBTQ individuals. We think that the findings on task-allocation and illegitimate-tasks call for more research in this subject, especially regarding the role of illegitimate tasks and task-allocation processes for the career development of women and the LGBTQ.

#### Lavender Over the Glass Ceiling

It is important to evaluate if, when, and what kind of leadership positions are available for gender minorities in organizations. This includes spotting cases when a single person or a small group is tokenized and expected to compensate for a lack of diversity of the whole organization (see Benschop and Doorewaard, 1998). The glass ceiling in the case of women and lavender ceiling in the case of LGBTQ individuals refer to the burdens faced by these groups to reach leadership positions as a consequence of sexism in organizations (Hill, 2009; Ezzedeen et al., 2015). There is also evidence that female executives are appointed to leadership positions when odds of failing are high (Ryan and Haslam, 2005). Regarding the LGBTQ, it is necessary to raise more evidence on the factors that make it possible for them to break through the lavender ceiling (Gedro, 2010).

### Limitations of This Study and Future Research

Our model was developed based on the review of available literature. The fact that it is based on secondary sources leaves space for bias and calls for its empirical testing. The mediation path that links the antecedents and consequences of GBDH should be tested in longitudinal studies, and the moderations proposed can be better assessed utilizing experimental designs. In this paper we argued for an integrative conception of gender in the HRM approach to GBDH. Nevertheless, data on the experiences of the LGBTQ in the workplace are mostly based on small samples, especially for the transgender. In addition, although we discussed the constructed nature of categories and pointed to their limitations, we considered women and the LGBTQ as relatively stable concepts. The experience of women and the LGBTQ greatly differs when looking to the heterogeneity between and within these groups. We thematized intersectionality mostly referring to sex assigned at birth, gender identity, and sexual orientation, and thus acknowledge our difficulty to account for exclusion dynamics involving identities in the intersection of race, gender, ableness, body form, and class. More research that focuses on these groups (e.g., transgender people of color) is needed. Finally, we made conjectures on the role that task-allocation processes may play as disguised GBDH that needs to be tested empirically as well. We think that since overt expressions of GBDH are in the decline in western workplaces, it is necessary to reach for gendered practices that disadvantage women and the LGBTQ in organizations.

## Conclusions

There is a potential for synergy when HRM considers the needs of women and people from the LGBTQ community together, especially to propitiate gender equality and counteract gender-based discrimination and harassment. To start, organizational resources can be employed to neutralize the mechanisms through which gender oppression acts against women and members from the LGBTQ community. In this way, actions for gender equality help create safe spaces for both groups. In addition, framing gender and sexuality in inclusive ways helps dismantle heterosexist, cissexist, and monosexist paradigms that contribute to create discriminatory and harassing workplaces. Finally, queer and feminist perspectives should be integrated with the intersectional approach to counteract discrimination against those in the intersection of multiple marginalized identities. Hence, the needs of people of all genders, people of color, disabled people, people with different body shapes, and people with different cultural backgrounds are made visible and addressed. This assists in developing truly inclusive and respectful workplace environments in which workers can feel safe to be themselves and unleash their full potential.

## Author Contributions

All authors contributed to the definition of the subject and the development of the hypotheses and model presented. CG drafted the manuscript and KO provided close support and supervision during the writing process and conducted revisions at all stages of the manuscript development. All authors contributed to the manuscript revision and approved the submitted version.

### Conflict of Interest Statement

The authors declare that the research was conducted in the absence of any commercial or financial relationships that could be construed as a potential conflict of interest.
